# FPGA Techniques Based New Hybrid Modulation Strategies for Voltage Source Inverters

**DOI:** 10.1155/2015/490151

**Published:** 2015-03-04

**Authors:** L. U. Sudha, J. Baskaran, S. A. Elankurisil

**Affiliations:** ^1^Department of Electrical and Electronics Engineering, ES Engineering College, Villupuram 605602, India; ^2^Department of Electrical and Electronics Engineering, Adhiparasakthi Engineering College, Melmaruvathur 603319, India

## Abstract

This paper corroborates three different hybrid modulation strategies suitable for single-phase voltage source inverter. The proposed method is formulated using fundamental switching and carrier based pulse width modulation methods. The main tale of this proposed method is to optimize a specific performance criterion, such as minimization of the total harmonic distortion (THD), lower order harmonics, switching losses, and heat losses. The proposed method is articulated using fundamental switching and carrier based pulse width modulation methods. Thus, the harmonic pollution in the power system will be reduced and the power quality will be augmented with better harmonic profile for a target fundamental output voltage. The proposed modulation strategies are simulated in MATLAB r2010a and implemented in a Xilinx spartan 3E-500 FG 320 FPGA processor. The feasibility of these modulation strategies is authenticated through simulation and experimental results.

## 1. Introduction

Pulse width modulation (PWM) strategies are dedicated for power converters to shape the output profile in a way beneficial to the industrial requirements. There have been several control strategies developed to enhance the fundamental component and minimizing the total harmonics distortion (THD) through elimination of lower order harmonics and to create a wider dead band for a given switching frequency [1–3]. Several attempts have been made in the last few decades to develop PWM schemes that differ in their concept and performance [4–8]. In 1999, an analog-based modified PWM control scheme has been developed to eliminate low order harmonic components caused by the nonconstant dc voltage [9]. In 1981, a method has been enabled to obtain the inverter output waveform variable continuously from a nearly sinusoidal to square wave and aimed at increasing the amplitude of the output fundamental without further adverse effects [10]. Harmonic elimination using Walsh function has been presented in [11] to cover optimized switching angles by solving linear algebraic equations instead of solving nonlinear transcendental equations. The THD optimization pattern has been reported in [12–14] presenting a method to eliminate the selected harmonics.

This paper corroborates three different hybrid modulation methods suitable for single-phase voltage source inverter derived using concatenation of fundamental switching and carrier based pulse width modulation (PWM) methods like unipolar PWM, inverted sine carrier PWM, and sixty-degree PWM, respectively. Consequently, with better harmonic profile for a target fundamental output voltage, the harmonic pollution in the power system will be moderated and the power quality will be improved.

## 2. Traditional PWM Methods

**Figure 1:**
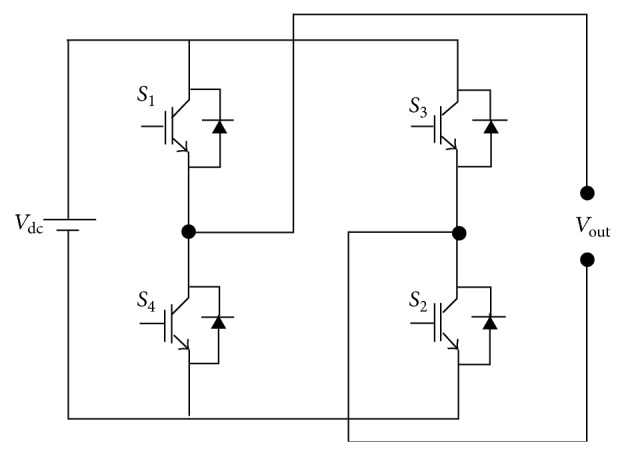
Single-phase inverter.

The PWM schemes developed for single-phase inverters shown in Figure 1 are used to improve the output profile through proposing carrier and reference modifications. Based on the detailed study, few of the traditional PWM strategies are discussed in this section. In unipolar switching scheme, the output voltage level changes either between 0 and −*V*_*d*_ or from 0 to +*V*_*d*_. This scheme “effectively” has the effect of doubling the switching frequency as far as the output harmonics are concerned, compared to the bipolar-switching scheme.

Similar to unipolar PWM, the voltage switches between two levels −*V*_*d*_ and +*V*_*d*_; the scheme is called the bipolar PWM. In this scheme the diagonally opposite transistors are turned on or turned off at the same time. For a full-bridge inverter with bipolar PWM scheme the output voltage is between −*V*_*d*_/2 and *V*_*d*_/2.

The inverted sine carrier PWM (ISCPWM) control scheme for single-phase full-bridge inverter eliminates poor output voltage spectral quality and reduced fundamental output voltage. It enhances the fundamental output voltage particularly at lower modulation index ranges while keeping the total harmonic distortion (THD) lower without involving changes in device switching losses.

The 60-degree PWM is similar to the modified pwm. The idea behind 60-degree PWM is to “flat top” the waveform from 60 degrees to 120 degrees and 240 degrees to 300 degrees. The power devices are held on for one-third of the cycle (when at full voltage) and have reduced switching losses. The 60-degree PWM creates a large fundamendal (2/3) and utilizes more of available dc voltage than does sinusoidal PWM. The output waveform can be approximated by the fundamental and the first few terms.

## 3. Proposed Hybrid Modulation Methods

**Figure 2:**
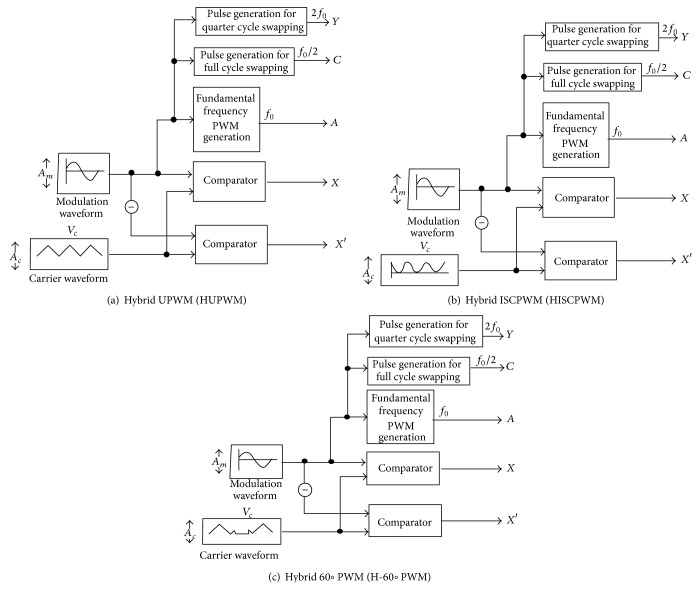
Proposed hybrid strategies.

The main philosophy of the PWM modulation emphasizes providing desired fundamental magnitude and reduced THD by pushing the lower order harmonics to higher carrier band thereby minimizing the filter design. In this direction, a new PWM strategy is developed to offer lesser switching loss with improved harmonic profile while offering desired fundamental output voltage. The proposed hybrid pulse width modulation (HPWM) strategies shown in Figure 2 are the amalgamation of fundamental frequency switching (FFS) and carrier based PWM (CBPWM), as a result that the output pattern acceded to the texture of switching-loss reduction from FFS and better harmonic profile from CBPWM.

The hybrid switching controller (HSC) basically consists of logic circuits generating gating signal for each device with two different frequencies, quarter cycle being switched at FFS, while the other quarter cycle is modulated at CBPWM. A random switching scheme is embedded with this hybrid modulation in order to swap both FFS and CBPWM for every quarter cycle of the output waveform. A simple base PWM circulation scheme is also introduced here to get resultant hybrid PWM circulation that inherits the characteristics of CBPWM methods. The hybrid modulation scheme consists of base PWM circulation and hybrid switching controller (HSC) to generate new modulation pulses.

The block diagram representation of base PWM circulation design is seen in Figure 2. It is basically a PWM generation (*X* and *X*′) acquired from a direct comparison of reference sine and carrier waveforms. Based on the selection of carrier, the PWM generation is classified as hybrid unipolar pulse width modulation (HUPWM) obtained from the comparison between sine reference and unipolar carrier, while hybrid inverted sine carrier pulse width modulation (HISCPWM) for inverter module is generated from the inverted sine carrier. The other PWM method reported for fundamental enhancement with improved harmonic profile, namely, H-60° PWM (HSDPWM).

In addition to PWM circulation pulses, the HPWM requires three square wave signals that make every power switch operating at carrier based PWM (CBPWM) and square wave randomly per quarter cycle to equalize the power losses within each device. Two signals are random switching pulse (*C* and *Y*) out of three square wave signals having 50% duty ratio and frequencies with half and twice the fundamental frequency. The third signal is FFS (*A*), a square-wave signal synchronized with the reference sine waveform, (*A* = 1) during the positive and (*A* = 0) during negative half cycles of the reference sine.

HSC combines random signals (*Y* and *C*), FFS (*A*), and CBPWM (*X* and *X*′) that produces hybrid modulation (HM) pulses. It is designed by using a simple combinational logic and the functions for a single-phase inverter are expressed as

(1)  S1=Y·C+Y¯·C¯·A+X,

(2)  S2=Y·C¯+Y¯·C·A+X,

(3)  S3=Y·C¯+Y¯·C·A¯+X′,

(4)  S4=Y·C+Y¯·C¯•A¯+X′.

HSC controller constituted using equations (1) to (4); each gate signal is composed of both low and high frequency signals. If *A* = 1, *S*_1_ and *S*_2_ are commutated at low frequency in the first and second quarter of first half cycle, while both devices are modulated with PWM in the second and first quarter cycles in the same half cycle of fundamental frequency, respectively. Similarly if *A* = 0, *S*_3_ and *S*_4_ are modulated with the same pattern in the second half cycle of the fundamental frequency as those in the devices *S*_1_ and *S*_2_, respectively. Therefore voltage stress and current stress of four switches are also equalized. The proposed HPWM method allows use of similar power devices with identical heat sinks, thus permitting a more reliable design.

## 4. Simulation Results

In order to evaluate the quality of output voltage waveform acquired from single-phase inverter using the proposed HPWM methods, the simulation study is carried in MATLAB/Simulink platform. The input parameters for simulation study are taken with dc-link voltage (*V*_dc_) = 200 V, switching frequency of PWM signal (*f*_*s*_) = 1 kHz, fundamental frequency (*f*_0_) = 50 Hz, *R* = 150 Ω, and *L* = 100 mH, respectively.

**Figure 3:**
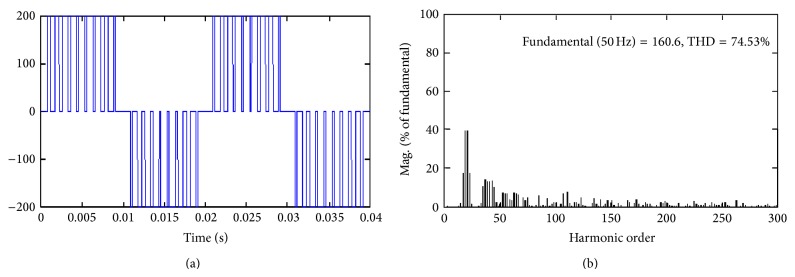
Proposed Hybrid UPWM (HUPWM).

**Figure 4:**
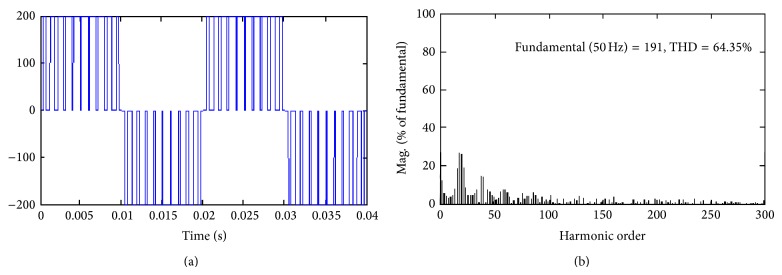
Proposed Hybrid ISCPWM (HISCPWM).

**Figure 5:**
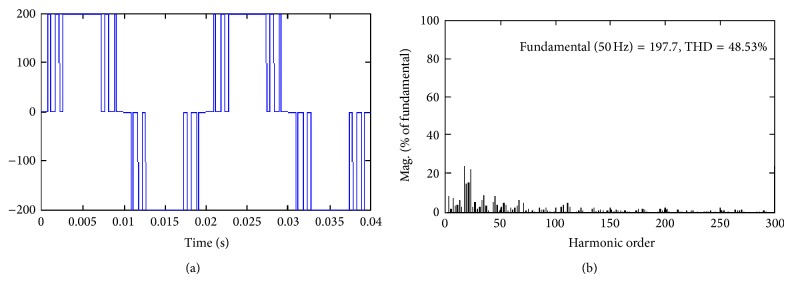
Proposed Hybrid 60° PWM (H-60° PWM).

Thorough simulation study has been carried out for single-phase inverter by keeping *M*_*a*_ = 0.8 for all the proposed hybrid modulation methods. It is evident from Figures 3 to 5 result that HUPWM produces 191 V (peak) with THD of 64.35% and HISCPWM offers fundamental voltage of 197.7 V (peak) with THD of 48.53%.

**Figure 6:**
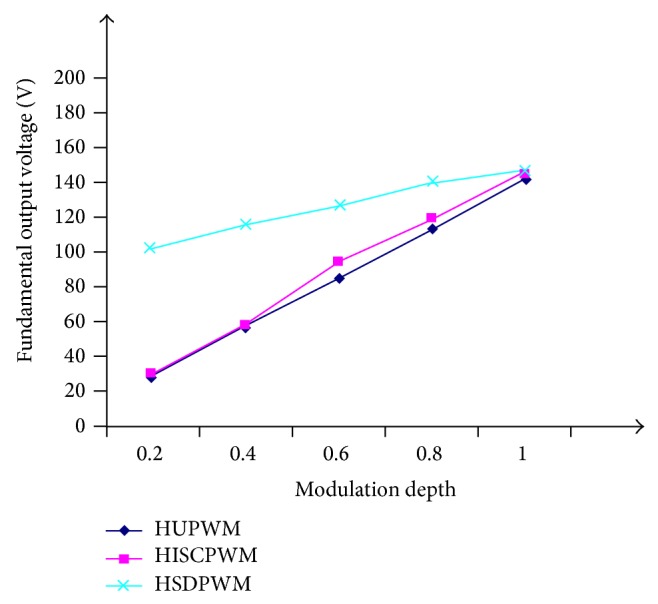
Fundamental output voltage against modulation depth.

Figure 6 shows the variation of fundamental output voltage against modulation depth. It is authenticated that the fundamental voltage goes on increasing as the modulation depth approaches 1. HSDPWM have more fundamental output voltage compared with HUPWM for a given modulation depth as depicted in the same Figure 6 and similarly it is seen that the H-60° PWM offers lesser THD with increase in modulation depth.

## 5. Experimental Results

**Figure 7:**
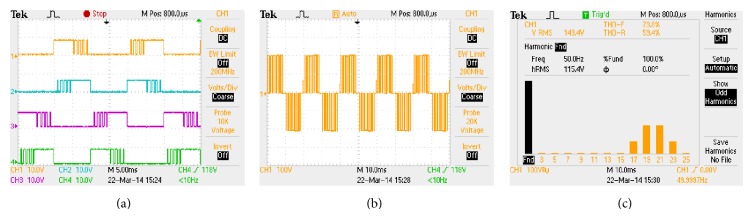
HUPWM (a) pulse pattern, (b) output voltage waveform, and (c) harmonic spectrum.

**Figure 8:**
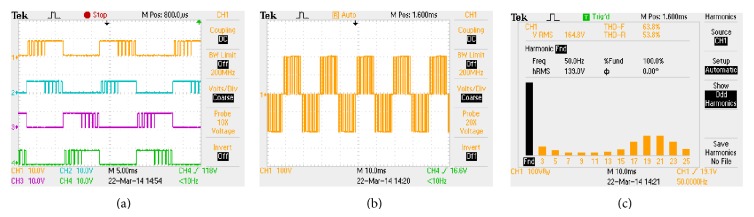
HISCPWM (a) pulse pattern, (b) output voltage waveform, and (c) harmonic spectrum.

**Figure 9:**
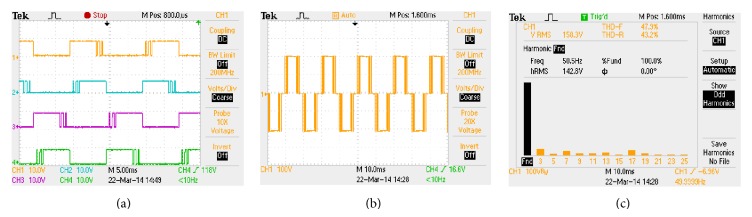
HSDPWM (a) pulse pattern, (b) output voltage waveform, and (c) harmonic spectrum.

The hardware rig is constituted using MOSFETs (IRF 840), laboratory variable dc-link voltage setup and resistive-inductive load of *R* = 100 Ω and *L* = 100 mH. The control algorithm is implemented in Xilinx Spartan 3E-500 FG 320 field programmable gate array (FPGA) processor. It incorporates a facility to be either configured or reconfigured by the application engineer. The Spartan 3E-500 FG 320 has on-board high-speed USB port, 500 K logic gates, 16 MB of PSDRAM, 16 MB of Intel strata flash ROM, and 60 FPGA input/output routed to expansion connectors. Xilinx offers CAD tools for the development of ASIC based FPGA and VHDL is used in digital logic design. The algorithm is then complied and simulated using Modelsim software. The bit file is generated in Xilinx software and finally downloaded to the Spartan 3E device through USB cable. The downloaded pulses are applied to single-phase inverter to produce the PWM modulated output voltage waveform. The download pulses, load output voltage waveform along with voltage spectrum, are captured through Tektronix TPS 2024 scope which is shown in Figures 7 to 9, respectively. It is evident from Figures 7 to 9 that the simulation outputs accorded with the experimental results.

## 6. Conclusion

Various HPWM strategies for single-phase inverter operating at low switching frequency with lesser switching commutations are proposed. The proposed HPWM method can be extended to any carrier based PWM methods. In comparison with conventional PWM methods, the proposed methods offer lesser number of switching commutations and reduced switching loss for the same fundamental voltage. The harmonic performance of the HPWM methods are examined in the entire operating range and its performance is better. The proposed methods facilitate balanced conduction loss with reduced switching loss within each device. Also the hybrid PWM strategy reduces conduction losses to a larger extent so that the THD is better when compared to the conventional PWM methods. The experimental results demonstrate that the proposed methods can be extended to any type of dc-ac/ac-ac power conversion systems.
